# Nursing and midwifery students’ attitudes towards principles of medical ethics in Kermanshah, Iran

**DOI:** 10.1186/s12910-019-0364-z

**Published:** 2019-04-25

**Authors:** Haleh Jafari, Alireza Khatony, Alireza Abdi, Faranak Jafari

**Affiliations:** 1 School of Nursing and Midwifery, Tehran University of Medical Sciences, Tehran, Iran; 2 Social Development and Health Promotion Research Center, Kermanshah University of Medical Sciences, Kermanshah, Iran; 3 Students Research Committee, Kermanshah University of Medical Sciences, Kermanshah, Iran; 4 Clinical Research Development Center of Imam Reza Hospital, Kermanshah University of Medical Sciences, Kermanshah, Iran

**Keywords:** Attitudes, Midwifery, Nursing, Professional ethics, Students

## Abstract

**Background:**

Professional ethics is one of the important topics, which includes various rights such as respecting the patient’s right to choose (autonomy), being useful (beneficence), being harmless (non- maleficence), and respecting the justice, integrity, and confidentiality of information. Adherence to these principles can increase the quality of care and patient satisfaction. Since determining the current attitude of students towards ethics plays an important role in educational programs, this study was conducted to evaluate the attitude of nursing and midwifery students of Kermanshah University of Medical Sciences towards six principles of professional ethics.

**Methods:**

In this cross-sectional study, 76 undergraduate nursing and midwifery students (who were at the final years of their study) were selected to participate in this study by census method. The data collection tool was a valid and reliable questionnaire on the principles of medical ethics. Data were analyzed using descriptive and inferential statistics.

**Results:**

The study sample consisted of 49 (64.5%) nursing and 27 (35.5%) midwifery students with an average age of 23 ± 1.4 years. The mean score of nursing and midwifery students’ attitude towards medical ethics was 95.01 ± 4.8 in basis of 100, and was 94.56 ± 4.9 for nursing students and 96.04 ± 4.7 for midwifery students. Majority of the samples (96.26%) had positive attitude towards medical ethics and 3.73% had a relatively positive attitude. No statistically significant relationship was found between the attitude of students and variables of gender (t = − 0.27, *p* = 0.78), field of study (t = − 1.3, *p* = 0.99), marital status (t = − 1.378, *p* = 0.178), and age (F = 1.606, *p* = 0.2).

**Conclusion:**

All students in this study had a positive attitude towards the principles of medical ethics, and this is a valuable asset for clinical environments. To increase the generalizability of the study, further studies with bigger sample size on the students of various disciplines of medical sciences is recommended.

## Background

Ethics is defined as the set of moral principles that form the basis of individuals’ behavior, and in a narrower sense, is the science of “correct” and “incorrect” behaviors, which is divided into theoretical and applied categories. Theoretical ethics is related to the meaning and objectives of morality and examines the fields of responsibility, but applied ethics is helpful in the right and wrong decision makings [1]. Professional ethics, which attempts to apply moralities in the health professionals’ practice as well as the field of ethical decision making in medicine [2], is one of the new branches of applied ethics that tries to respond to the variety of professional ethical issues through certain principles [3], according to which, medical practitioners and nurses should provide high-quality care based on professional and ethical standards [4]. Applied ethics helps healthcare professionals to treat each patient according to his/her values ​​and prevent him/her from harm [1]. In other words, professional ethics is an analytical activity in which thoughts, beliefs, obligations, behaviors, feelings, reasoning, and arguments in the field of ethical decision makings in medicine are carefully and critically examined, and if necessary, guidelines are issued and values, rights or wrongs, corrects or incorrect in the field of health performance and care are addressed [5].

Florence Nightingale’s commitments can be considered as the first ethical codes that provided new professional values for nursing profession and caused nursing to be viewed as a profession with unique values and principles. Subsequently, the American Nursing Association (ANA, 1950) and the International Nursing Council (ICN, 1953) reviewed existing codes and presented new codes of Nursing Ethics [1]. The reason for the philosophy and the topic of medical ethics and the emphasis on its importance is that, these ethics are beyond the general ethics and what is considered as general ethics cannot meet the ethical needs of medical and allied health professions [6]. Recent advances in medical sciences and technology have added to the complexity of nurses’ practice and today, nurses are faced with many moral problems [7] and ethical issues, which make ethical decision making very difficult in medical terms. It seems that, traditional medical ethics is no longer able to respond to these new needs, so we need to revitalize, promote, and teach professional ethics [8]. Since the performance of nurses as the largest provider of services in the healthcare system has a significant impact on the quality of care, compliance to ethical standards is an effective factor for improving the nurse’s performance to provide quality of care [9]. However, compliance to professional ethics is not limited to nurses and includes all healthcare professionals, such as physicians, nurses, midwives and other members of the healthcare multidisciplinary team who encounter ethical challenges in their work activities. Everyone agrees that, having ethical sensitivities is one of the essential requirements for their performance [10]. In this way, high quality and non-judgmental care without discrimination should be provided to all patients, and respecting human rights and treating individuals, regardless of their position should be considered as an integral part of the healthcare system [11].

Professional ethics includes four main general principles, including autonomy, beneficence, non-maleficence, and justice (1.12), and autonomy itself includes several principles, such as integrity and privacy of the patient [1]. Teaching these principles to the members of healthcare multidisciplinary team is one of the requirements of professional ethics [2] and it is expected that physicians and nurses be aware of these principles and consider them in their practice [12]. Thus, in recent years, with the increase of issues related to medical ethics, the promotion of professional ethics among various students of medical sciences, especially those who are in direct contact with patient, has become very important [13].

Despite the importance of professional ethics in medical sciences, evidence of unethical behaviors by medical and nursing students in different environments has been reported [12]. In some studies, many of the participants believed that, there was insufficient knowledge and awareness among young students and nurses about the appropriate ethical performance, because of an assumption that, there is a direct relationship between having knowledge about values and professional ethics, and ethical behavior of nurses [14]. According to some studies, many physicians and nurses were unaware of major documents of health care ethics such as Helsinki, and most of them considered lectures, books and magazines as the main sources of ethical information [12, 15, 2]. In this regard, the results of a study conducted on the nursing students of Guilan University of Medical Sciences in Iran showed that, most of the students were not aware of the codes of nursing ethics [15]. Motamed Jahromi (2014) also emphasized on the importance of professional ethics and its application in clinical settings, and highlighted the need for education in the field of medical ethics and provision of workshops to teach these principles to students in order to familiarize them with the ethical challenges that exist in clinical settings and the ways to address them [2]. According to many studies, in addition to ethical education, students’ active participation in discussions and the use of a specific ethical framework [16], the presence of tutors as role models can also enhance students’ ethical performance [14]. Caldicott (2009) emphasizes on the importance of professional ethics and suggests that, ethical theories such as Kant as well as various educational modules on ethics and physician-patient communication should be used with the aim of achieving desired health objectives [17]. In Iran, in order to promote professional ethics among undergraduate nursing students, a lesson is offered entitled “Professional Ethics in Nursing” in the form of 1.5 credit, and a lesson is provided for midwifery students, under the title of “Ethics and regulations of midwifery” in the form of 1 credit.

Although ethics and professional values are global issues, they can be influenced by culture as an important factor [18], and despite the importance of teaching professional ethics and its impact on employee’s performance, it could be harmful if unique social and economic features as well as geographic location are not carefully considered. Hence, educational lessons on professional ethics should be taught by taking into account the appropriate social and cultural backgrounds. To formulate educational modules in ethics in each region, the first step may be to determine the current attitude of people in the region towards ethics [19]. Therefore, considering the role of professional ethics in preserving the dignity of patients on one hand and the medical staffs on the other hand, as well as improving working conditions and increasing the positive effects of healthcare services, it is necessary to determine and explain the principles of professional ethics among students of various disciplines of medical sciences in different societies and cultures. In this regard, the present study was conducted to investigate the attitude of nursing and midwifery students of Kermanshah University of Medical Sciences towards the six principles of professional ethics.

## Materials and methods

### Research question

What are the attitudes of nursing and midwifery students of Kermanshah University of Medical Sciences towards the six principles of professional ethics?

### Research design

The present analytical descriptive study was conducted during the second educational semester (2013–2014) at the Nursing and Midwifery Faculty of Kermanshah University of Medical Sciences (KUMS). The Faculty of Nursing and Midwifery in Kermanshah has more than 50 years of history, and 373 nursing students and 152 midwifery students studying there. Nursing and midwifery students use similar physical spaces and facilities as other students of the university, but depending on their educational objectives, their internship sections may be different.

### Sample and sampling method

The study population consisted of all final year nursing and midwifery students (7th and 8th semesters) at the second semester of the academic year 2013–2014, that were selected by census sampling method. Among the 105 students who participated in the study, 64 were nursing and 41 were midwifery students. In total, 72.3% of the participants completed the questionnaires, and among the 105 distributed questionnaires, 29 had not been completed properly and only 76 of them were considered in the study, which belonged to 49 (64.5%) nursing students and 27 (35.5%) midwifery students.

The criteria for entering the study included; to be in the last year of study (7th and 8th semesters) at bachelor’s degree in nursing or midwifery, and to give written consent for participation in the study. Samples were excluded from the study if they had not completed the questionnaires properly.

### Measurement instrument

The data gathering tool was a two-part questionnaire. The first part was devoted to examining the personal characteristics of the samples (age, gender, marital status, and field of study) with 4 questions. The second part of the questionnaire was dedicated to the students’ attitudes toward six principles of professional ethics, including principles of respecting patient autonomy, beneficence, non-maleficence, justice, honesty and respecting patient’s confidentiality. The Professional Ethics Questionnaire was developed by Motamed Jahromi and Dehghani (2014) based on their previous studies on this issue, which aimed to assess the students’ attitudes toward the principles of medical ethics in Iran. The validity and reliability of the questionnaire have been reviewed and approved. For this purpose, content validity method was used to assess the validity, and Cronbach’s alpha method was estimated to evaluate the reliability of the questionnaire (alpha coefficient was 0.75) [2]. In our study, the content validity of the questionnaire was reviewed by 10 experts in order to correct the final verifications of the items. The reliability of the questionnaire was also re-evaluated using Cronbach’s alpha, and the alpha coefficient of 0.82 was obtained. The questionnaire included 18 questions (three questions for each of the six principles of medical ethics mentioned above) based on a 5-item Likert scale, including completely agree [5], relatively agree [4], no comment [3], relatively disagree [2] and completely disagree [1]. The minimal score was 18 and a maximum score was 90. By calculating the scores obtained from the questionnaire in the basis of 100, the score of 75 and above was considered a completely positive attitude, 51–54 score reflected a relatively positive attitude, score of 50 reflected a neutral attitude, score of 25–59 meant a relatively negative attitude, and a score of less than 24 reflected a negative attitude.

### Data collection

From March 2013 to May 2014, Considering the educational schedule of students, the researcher attended the faculty and hospitals and conducted the sampling. For this purpose, the objectives of the study were first explained to the samples and the necessary reassurances regarding the confidentiality of personal information and how to respond to the questions were given to them and a written consent form was obtained from the samples. After signing the consent form, the questionnaires were completed by the students and collected by the researcher.

### Data analysis

Data were analyzed by 18th version of the Statistical Package for Social Sciences (SPSS v.18.0; SPSS Inc., Chicago, IL, USA), using descriptive (frequency, mean, standard deviation) and analytical (Chi square, independent t, one-way ANOVA) statistics and *P* < 0.05 was considered as the significance level.

### Ethical consideration

Ethical Review Committee of KUMS approved the study with the code IR.Kums.rec.1397.1058. The written informed consent was obtained from the participants. All of the Participants were also assured of the confidentiality of their personal information.

### Findings

Based on the findings of this study, majority of the samples were female (56 students, 73.7%), single (64 students, 84.2%) and nursing students (49 students, 64.5%). The mean and standard deviation of age was 23 ± 1.4 years and most of them (48 students, 63.2%) were 21–23 years old. The average score of students’ overall attitude towards professional ethics in the basis of 100 was 95.01 ± 4.8, and this score was 94.56 ± 4.9 for nursing students and 96.04 ± 4.7 for midwifery students. Majority of the samples (96.26%) had positive attitude and 3.73% of the samples had a relatively positive attitude towards medical ethics. No significant relationship was found between the attitude of students and variables of gender (t = − 0.27, *p* = 0.78), field of study (t = − 1.3, *p* = 0.199), marital status (t = − 1.378, *p* = 0.178), and age (f = 1.606, *p* = 0.2), (Table 1).

**Table 1:** Comparing the mean scores of attitudes toward principles of medical ethics based on demographic characteristics

variables		Mean ± SD	n (%)	Test result
Gender	Male	94.8 ± 4.9	20 (26.3)	*P* = 0.789
	Female	95.2 ± 4.8	56 (73.7)	t = −0.27
Field of study	Nursing	94.56 ± 4.9	49 (64.5)	*P* = 0.199
	Midwifery	96.04 ± 4.7	27 (35.5)	t = −1.3
Marital status	Single	94.9 ± 5.1	64 (84.2)	*P* = 0.178
	Married	96.2 ± 2.9	12 (15.8)	t = −1.378
Age (Yrs.)	21–23	94.1 ± 0.74	48 (63.2)	*P* = 0.2
	24–26	96.5 ± 0.65	25 (32.9)	
	≤ 27	94.4 ± 0.8	3 (3.9)	F = 1.606

The results also showed that, in relation to each of the six principles of medical ethics, including respecting patient’s autonomy, beneficence, non-maleficence, justice, honesty, and confidentiality, most of the samples had a positive attitude and some had a relatively positive attitude, but none of the students had negative, relatively negative or even neutral attitudes towards these principles (Table 2).

**Table 2:** The distribution of the mean scores of the attitudes of the nursing and midwifery students toward principles of medical of ethics (N: 76)

Medical ethics principles	Attitude dimensions				
	positive	relatively positive	neutral	relatively negative	Negative
Autonomy	75 (98.7)	1 (1.3)	**___**	**___**	___
Non- malfeasance	73 (97.3)	2 (2.7)	**___**	**___**	___
Beneficence	75 (98.7)	1 (1.3)	**___**	**___**	___
Justice	74 (97.4)	2 (2.6)	**___**	**___**	___
Veracity	66 (86.8)	10 (13.2)	**___**	**___**	___
Confidentiality of information	75 (98.7)	1 (1.3)	**___**	**___**	___

## Discussion

The purpose of this study was to determine the attitude of nursing and midwifery students towards the six principles of professional ethics. In this regard, the results showed that, most students had a positive attitude towards professional ethics (96.26%), and if each principle was to be examined separately, it would appear that, almost all participants had positive attitudes toward each of the principle, and only 13.2% of the samples had a relatively positive attitude towards the principle of Veracity. In a study to assess the perception and performance of medical professionals in selected hospitals in Malaysia and India by Yousuf (2007), 42% of the samples in one hospital and 44% in another hospital believed that; “If it is considered harmful, I will still break the news” [20]; Like the present study, samples of this study also suggest that some issues should not be told to the patient. In addition, similar studies have been conducted in this regard that might have focused on one ethical principle than others, but they have nevertheless highlighted the importance of this issue from the perspective of healthcare professionals. For example, in a study of 140 nurses in Turkey in 2015, almost all nurses stated that, they were trying to ensure the safety of patients, respect their autonomy, and prevent them from harm and possible dangers. In addition, 84.3% of nurses stated they do not prioritize their relatives and friends over others, which indicated their respect for the principle of justice, and 90% said they do not delay the care of disrespectful patients and treat them the same as others. The majority of nurses in this study (95.7%) also considered the privacy of the patient, and the results indicated that, nurses pay attention to the professional ethics [1]. In another study conducted on 128 physicians in Egypt in 2012 using a questionnaire and a checklist, nearly all physicians believed that, issue of medical ethics is important and majority of them (91.4%) could point to at least one of the ethical principles, including personal privacy (89.8%), informed consent (87.5%), honesty (85.9%) and beneficence (84.4%). More than two-thirds of the samples (68.7%) referred to non-maleficence, less than two-thirds referred to the patient’s autonomy and 4.59% of the samples referred to the principles of justice and confidentiality [21]. In another study that investigated the nurses’ professional values ​​in Iran in 2017, the patient’s confidentiality was the most important issue from the viewpoint of nurses [22].

The overall result of the present study regarding the students’ positive attitude towards professional ethics is in line with the Motamed Jahromi’s study that aimed to investigate the attitude of Kerman University of Medical Sciences’ students towards ethical principles and its adaptation to Islamic ethics. In the present study, this attitude was completely positive in all 6 principals, while in the study of Motamed Jahromi, students’ attitudes towards all principles was reported as completely positive except the principles of justice and honesty, which the attitude was reported to be relatively positive [2]. This positive attitude is considered to be an important asset for clinical environments, and authorities should do their best to maintain this positive attitude of student during practice at patient’s bedside. It seems that, working in clinical setting has a tendency to become routine for healthcare staffs and due to their high workload, some aspects of medical ethics may be ignored by them. In this regard, Borhani et al. has reported a significant positive correlation between ethical distress and professional stress among nurses [23]. The study of Kayhani et al. that aimed to investigate the attitude of nurses of Gilan University of Medical Sciences towards medical ethics showed that, only 12.5% ​​of the samples had positive attitude towards professional ethics, 37.5% had poor attitude, and 60% had moderate attitude towards medical ethics [24]. Therefore, it is suggested that more emphasis should be placed on promoting the professional ethics on the patient’s bedside and some educational courses should be designed in this regard. A notably point in this regard is to encourage the nursing staff to participate in these courses and also information on the provision of these courses should be widely available to everyone, because the result of Hariharan’s study indicated that, 29% of physicians and 37% of nurses were unaware of the existence of professional ethics committees in hospitals, and more than 70% of physicians and nurses stated that, they acquire ethical knowledge during work [19].

In the present study, there was no statistically significant relationship between students’ attitude and demographic variables including gender, age, field of study, and marital status. Karampourian investigated the attitude of 30 faculty members of Hamadan Faculty of Dentistry and obtained similar results [3], but the study of Motamed Jahromi showed that, the attitude of students over 20 years old was more positive than the attitude of students who were less than 20 years of age. He also showed that, the attitude of married students, male students, and medical students was more positive than the attitude of single students, female students, and students of other disciplines of medical sciences [2]. In the study by Mokhtari Lakeh et al. female students had a more positive attitude towards nursing ethics than male students, and this difference was statistically significant [15]. The lack of significant statistical relationship between students’ attitude and demographic variables could be due to the relatively small sample size in these studies, and it may be possible to observe significant statistical differences by increasing the sample size.

### Study limitations

The relatively small sample size of this study could reduce the generalizability of its findings. Studying the attitude of students through self-reporting method could also overestimate the results of this research.

### Recommendation

In order to increase the generalizability of the findings, further studies should be conducted with a larger sample size and on students of different disciplines of medical sciences. Studies can also be conducted on a wider scale and compare the attitudes of students of different universities of medical sciences across the country, so that, the impact of environment and educational programs on students’ attitudes towards medical ethics could also be examined.

## Conclusion

Despite the fact that, all students in this study had a positive attitude towards the principles of medical ethics and no significant relationship was found between the attitude of students and variables of gender, field of study, marital and age. This positive attitude towards the principles of medical ethics is a valuable asset for clinical environments and we should improve it evermore.

## Abbreviations

ANA: American nursing association
ICN: International nursing council
KUMS: Kermanshah university of medical sciences
